# Complications of Extracorporeal Shock Wave Lithotripsy for Urinary Stones: To Know and to Manage Them—A Review

**DOI:** 10.1100/2012/619820

**Published:** 2012-03-12

**Authors:** Alessandro D'Addessi, Matteo Vittori, Marco Racioppi, Francesco Pinto, Emilio Sacco, PierFrancesco Bassi

**Affiliations:** Department of Urology, Catholic University School of Medicine, Policlinico “A. Gemelli”, Largo F. Vito, 00168 Rome, Italy

## Abstract

To identify the possible complications after extracorporeal shock wave lithotripsy (SWL) and to suggest how to manage them, the significant literature concerning SWL treatment and complications was analyzed and reviewed. Complications after SWL are mainly connected to the formation and passage of fragments, infections, the effects on renal and nonrenal tissues, and the effects on kidney function. Each of these complications can be prevented adopting appropriate measures, such as the respect of the contraindications and the recognition and the correction of concomitant diseases or infection, and using the SWL in the most efficient and safe way, tailoring the treatment to the single case. In conclusion, SWL is an efficient and relatively noninvasive treatment for urinary stones. However, as with any other type of therapy, some contraindications and potential complications do exist. The strictness in following the first could really limit the onset and danger of the appearance of others, which however must be fully known so that every possible preventive measure be implemented.

## 1. Introduction

Since its appearance at the beginning of the 1980s [1], extracorporeal shock wave lithotripsy (SWL) has been confirmed as the least invasive and the most widely used treatment of kidney and ureteral stones, also in acute conditions [2]. Naturally, like any other treatment, its efficacy is indeed accompanied by some side effects and complications that, despite being generally mild in nature, require accurate evaluation and implementation of measures to prevent them. An example is the flank pain during the procedure, which has not to be considered as a complication, but rather an undesired side effect to deal with very often and which can sometimes induce the patient to ask for the interruption of the treatment. The protocol of the procedure should so include an analgesic prophylaxis, and therapy with opioids or nonsteroidal anti-inflammatory drugs were both evaluated [3].

In essence, we are not talking about a procedure that is altogether benign, but rather one that may bring about lesions to kidneys and/or its neighbouring organs. Moreover, even a technically successful lithotripsy may determine subsequent morbidity due to related fragmented products. In light of this, the few contraindications that do actually exist should clearly be kept in mind [4]. These are:

- pregnancy;
- uncontrolled infections in the urinary system;
- uncontrolled alterations of coagulation;
- aortic or renal artery aneurism;
- serious skeletal malformations;
- serious obesity.

## 2. Classification

Complications after an SWL come from:

- the formation and passage of fragments;
- infections;
- the effects on renal and nonrenal tissues:
  - the effects on kidney function;
  - hypertension.

### 2.1. Complications Related to the Formation and Passage of Lithiasic Fragments

The main aim of an SWL is the pulverisation of stones and asymptomatic elimination of fragments. This procedure may not always be completely successful due to *incomplete fragmentation, *with residual fragments of a significant size, and ureteral blockage by fragments (Steinstrasse) which ends up with an obstruction to the urinary flow.

To illustrate this, the formation of fragments <4 mm is present in up to 59% of the cases, with a risk of a symptomatic episode, an operation, or even both, equal to 43% [5].

Factors responsible for the level of fragmentation after a lithotripsy, and therefore real risk factors for SWL failure are the *composition*, *volume, site, the number of stones, and the frequency and strength of the shock wave*.

#### 2.1.1. Composition

The stones made by Struvite, uric acid, and dehydrated calcium oxalate tend to fragment into tiny parts that may be easily passed. On the other hand, dehydrated calcium phosphate stones (brushite) and monohydrate calcium oxalate stones tend to produce larger fragments which are hence much harder to pass. Particularly difficult to treat are the stones made by cystine which, like any organic compound, has acoustic features similar to those in the surrounding tissues.

#### 2.1.2. Volume

The chance of SWL treatment success is related to the volume of the stones being treated. For stones <2 cm, the percentage of success reported, considered as “stone free rate,” has been in the range of 66–99%, which drops to 45–70% for stones of 2-3 cm and even further again for staghorn stones [1, 6–8].

Moreover, stones >2 cm almost always require multiple treatments and have a tendency to shatter incomplete: the risk of complication is greater with an incidence of partial obstruction between 19–50% [8]. In some cases, it is size alone that determines treatment: after an SWL, cystine stones <15 mm shatter in 71% of cases; if the size of the stone is >20 mm, the success rate drops to 40% [9].

For this reason, SWL as a monotherapy for cystine stones >15 mm is currently not recommended [4].

#### 2.1.3. Number and Site

The chances of success are less, all other characteristics being equal, for stones located in the lower pole of the kidney. Recorded success rates have been in the region of 29% for stones of 11–20 mm and 20% for stones >20 mm [10] that, moreover, often require multiple treatments to be cleared up. The presence of multiple stones has been tied to a larger number of relapses after SWL [1, 11]. For ureteral stones, the percentage of overall success is not as high in absolute terms and depends, above all, on the segment where the stone is located: proximal ureter 82%, medial ureter 73%, and distal ureter 74% [4].

#### 2.1.4. Frequency and Strength of Shock Wave

Although the effects of shock wave frequency on the efficacy of the treatment were not clinically widely evaluated [12], in vitro studies have shown that a reduction in frequency improves the possibility of fragmentation [13] and an increase in the voltage supplied is related to a reduction in lesser volume fragments [14]. In addition, the energy font used was compared to the results so, for example, an electrohydraulic lithotripsy supplied fragments <2 mm in 91% of the cases while an electromagnetic one did so in only 65% of the cases [15]. Furthermore, success rates of 63% and 83% were recorded for different models of the same energy font, even if other studies have not as of yet confirmed this difference in performance [16, 17].

One complication directly related to incomplete fragmentation is the pileup of fragments, otherwise known as *steinstrasse*. This complication appeared in 1–4% of patients, rising to 5–10% when the stone is >2 cm [18] and to 40% where staghorn stones were present [19]. At times, the complication resolved itself and with contained symptomatology, while on other occasions recurrent colics occur. To highlight any silent forms, the most insidious, a radiological or ultrasound examination should be carried out routinely 4–6 weeks after the SWL treatment. Stones of >3 cm should be treated percutaneously, however, where this is impossible, confirmation of steinstrasse following an SWL is a likely course of action and the placing of a ureteral stent may in this case reduce the incidence of piling up of fragments. Nevertheless, the presence of a stent does not reduce the incidence of Steinstrasse in the case of small-to-medium-sized stones, and should thus be avoided [18]. Also in the case of ureteral stones, a stent does not seem to be particularly useful [4].

Different options exist to deal with the problem once it has been established. As we have already seen, in some cases, complications are asymptomatic and may simply be followed over time with spontaneous resolution of the problem in 2 to 4 weeks, always ensuring of course that renal function is maintained [20]. Possible administration of medical treatment made up of associated *alpha*-blockers or even corticosteroids [21, 22] may accelerate the clearance of fragments. Where symptoms are present and the steinstrasse is no longer than 2.5 cm, it may still be a valid option to wait, that could resolve the complication in more than a half of the patients, naturally prescribing suitable pain control therapy.

In other cases, above all where larger distal fragments are present, steinstrasse has effectively been treated with repeated sessions of SWL showing positive results in 90% of cases [23]. With ureteral meatotomy results have also been satisfactory [24]. In more serious cases, where infections and complete obstructions are manifest, it is necessary to place a nephrostomy or to proceed with either a retrograde or anterograde percutaneous ureterorenoscopy.

### 2.2. Infective Complications

During extracorporeal lithotripsy, one of the forces applied to the stone comes from a cavitation bubble collapse. This force, however, may cause damage to the small renal vessels that would result in a microhaemorrhage, the release of cell mediator of phlogosis, and the infiltration by inflammatory response cells.

These tiny lesions may also allow the passage of bacteria, which may be present in the urine or inside the stones themselves, into the blood stream which could thus develop into other related problems. 

To simplify things here, we will define “infection” as a harmful colonization of a species unknown to the host organism that responds to the infection with inflammation. By the term “sepsis,” on the other hand, we refer instead to a serious medical condition characterised by a generalised state of inflammation, called SIRS (systematic inflammatory response syndrome), and by the definite or suspected presence of an infection [25]. Evidence of bacteriuria is present in up to 23.5% of patients [26], while a real clinical urinary infection is more frequently observed in patients with either multiple or complex struvite stones [27].

The development of sepsis after bacteremia is relatively low in absolute terms, <1% of cases [26], although it is considerably higher in the presence of staghorn stones [8]. The risk of infection is naturally greater, where urinoculture is positive or where urinary obstruction exists.

There are no truly trustworthy signs that attest to the early onset of bacteremia or bacteriuria: white cells blood count, speed of erythrosedimentation, and a positive culture are all useful signs, unfortunately they generally tend to show up positive when the patient is already symptomatic. In terms of reduction of infective complications and the expense connected to their treatment, the use of antibiotic prophylaxis has therefore been proposed, but this use has not been confirmed in other randomised controlled studies for patients without preexisting UTI or infected stones [28].

To sum up then, antibiotics should only be administered to patients with positive urine culture, with staghorn or low density struvite stones, with a history of struvite stones or recurring urinary infections, to patients who will undergo a contemporary instrumental procedure, and finally to those with a nephrostomy or a stent in place [27, 28].

### 2.3. Effects on Tissues

#### 2.3.1. Kidney

The most evident expression of kidney trauma is haematuria that generally passes in a few days.

Collections of symptomatic fluids or perirenal, subcapsular, or intrarenal haematomas are rare and occur in less than 1% of patients; if, however, patients have systematically undergone a CT scan or MRI then evidence of haematoma rises to 25% [29]. Other lesions show in X-rays in most patients: an increase in the volume in the kidney [30], a loss in corticomedullary demarcations [30], and a reduction in the signal in the perirenal fat. These signs express lesions such as haemorrhages, generally focalised, and oedema within and around the kidney [31]. Perirenal collections typically disappear after a few days, while a period of between 6 weeks and 6 months is required for subcapsular ones [32]. It is rare to see lesions for any longer than that. 

A microscopic examination shows up characteristic evidences: haemorrhagic lesions are preferentially localised in the corticomedullar joint, probably due to differences in the density of the tissue at that level [33]; moreover, signs of damage are immediately visible from the thin vascular walls and the glomeruli [34]. Haemorrhage leads to tissue hypoxia, which can play a role in the development of apoptosis, but it has been experimentally shown that shock waves administration does not affect the apoptosis index in normal rats after 2000 and 4000 shock waves [35] and after 1-2 weeks signs of reorganisation may be noticed, while after 1 month signs of glomerular atrophy and sclerosis are noticeable in tiny areas of fibrosis. However, most of the parenchyma appears normal [32], leading to the conclusion that damage due to SWL is a focal process that leaves most of the parenchyma intact. A short pretreatment with 10–20 shockwaves could further on reduce the renal tissue damage, probably due to a reflex local vasoconstriction.

#### 2.3.2. Cardiovascular Apparatus

The incidence of arrhythmia during an SWL varies from between 11% and 59%, and is, in general, related to minor premature ventricular beats. Evidence of ischemic lesions is very rare, and this incidence may be further reduced by synchronising the supply of shock waves with pulsations [36]. There is no documented relationship between the onset of arrhythmia and age, sex, cardiopathy, site, volume of the stone, onsite stent or nephrostomy, with or without anaesthesia, the number of shock waves, and the type of lithotripter [36]. Even those patients with pace makers may undergo an SWL with necessary precautions and cardiological supervision [37]. Although clinical and experimental data indicates that patients with aortic or renal aneurisms may be treated successfully, literature has reported some cases of breakage after an SWL [38]. It is clear, as in other similar cases, that a careful examination of the cost/benefit relationship is necessary and that where the procedure is embarked upon each and every possible development must be considered beforehand. Cases of serious venous thrombosis after SWL have also been recorded, the exact pathogenesis of which is still badly defined; however, it is probably caused by haematological disorders, even if this may just be to a small extent [39].

The association between SWL and arterial hypertension has always been a controversial argument and debated. The diagnosis of hypertension after SWL has been reported in 8% of cases, that does not differ greatly however from the incidence of about 6% of new diagnosis in the overall population [40]. An increase in diastolic pressure after an SWL was also noted, and a relationship between this and the number of shock waves was therefore hypothesised upon [41].

A large retrospective study has analysed patients who underwent an SWL, controlled against patients who underwent an ureterorenoscopy or a percutaneous lithotripsy without being able to show, within one year of the treatment, any significant differences in the incidence of hypertension (2.4% versus 4%), and even after 4 years, the differences were not particularly significant (2.1% versus 1.6%); however, a statistically significant increase in diastolic pressure showed up after SWL [42]. The real causes of hypertension after SWL are more likely to have many different factors, and there is no clear evidence whether there is any direct relationship between hypertension and the procedure even if one considers more recent studies that have demonstrated, with a followup of 24 months, how it is the presence of stones rather than the modality of treatment that determines the increase in pressure [43].

Many of the studies that have been documented are retrospective. Limiting oneself to randomly controlled studies there is no evidence that SWL treatment determines changes in arterial pressure [40, 44]; in fact, it is possible that the extracorporeal lithotripsy is responsible for hypotension, and likewise for alterations in renal metabolism determined by the treatment and function of the number and strength of shock waves administered [45].

#### 2.3.3. Gastrointestinal Apparatus

Several gastrointestinal lesions of various types have repeatedly been recorded following an SWL with a global incidence of 1.8% [46]. A specific study has shown gastroduodenal erosions in 80% of patients who underwent a pre- and post-SWL endoscopic study [47]. The exact mechanism of the lesions is, as of yet, unknown, however, the majority were observed in patients subjected to treatment in prone position and in patients who had undergone a number of shock waves that exceeds what is recommended [46].

#### 2.3.4. Fertility and Pregnancy

A sufficiently high amount of clinical and experimental evidence exists to exclude any permanent effects on testicular or ovarian function to thus confirm that there are no existing correlations between SWL and fertility [48, 49]. Pregnancy, however, constitutes an absolute contraindication to the procedure itself because of any potentially harmful effects to the foetus from shock waves, as has repeatedly been shown in the results of many experimental studies [50].

## 3. Conclusions

Extracorporeal lithotripsy is an efficient and relatively noninvasive treatment for urinary stones: the large number of cases treated using this procedure, and its widespread use, testifies to this. However, as with any other type of therapy some contraindications and potential complications do exist. The strictness in following the first could really limit the onset and danger of the appearance of others, which however must be fully known in order that every possible preventive measure be implemented.

## List of Abbreviations

SWL: Extracorporeal shock wave lithotripsy
SIRS: Systematic inflammatory response syndrome
UTI: Urinary tract infections
CT: Computerised tomography
MRI: Magnetic resonance imaging.
